# A Recommendation for a Pre-Standardized Marine Microalgal Dry Weight Determination Protocol for Laboratory Scale Culture Using Ammonium Formate as a Washing Agent

**DOI:** 10.3390/biology10080799

**Published:** 2021-08-19

**Authors:** Yam Sim Khaw, Hui Teng Tan, Arissara Sopawong, Noor Azmi Shaharuddin, Abdul Rahman Omar, Fatimah Md. Yusoff

**Affiliations:** 1 Laboratory of Aquatic Animal Health and Therapeutics, Institute of Bioscience, Universiti Putra Malaysia, Serdang 43400, Selangor, Malaysia; yskhaw@gmail.com (Y.S.K.); huiteng.tan28@gmail.com (H.T.T.); arissara.so@gmail.com (A.S.); 2 Department of Biochemistry, Faculty of Biotechnology and Biomolecular Sciences, Universiti Putra Malaysia, Serdang 43400, Selangor, Malaysia; noorazmi@upm.edu.my; 3 Laboratory of Vaccines and Immunotherapeutic, Institute of Bioscience, Universiti Putra Malaysia, Serdang 43400, Selangor, Malaysia; aro@upm.edu.my; 4 Department of Aquaculture, Faculty of Agriculture, Universiti Putra Malaysia, Serdang 43400, Selangor, Malaysia; 5 International Institute of Aquaculture and Aquatic Sciences, Universiti Putra Malaysia, Port Dickson 71050, Negeri Sembilan, Malaysia

**Keywords:** marine, microalgae, biomass, dry weight, best conditions, ammonium formate

## Abstract

**Simple Summary:**

Microalgae are increasingly recognized as a source of valuable biomass with numerous health benefits. Cleaning of marine microalgal biomass is very crucial for microalgal studies as the salt on the microalgae cells will lead to overestimation of biomass determination. Incomplete washing of salt from microalgae could also interfere with the nutritional analyses. The biomass, especially dry weight, has been utilized for nutritional or compositional evaluation. Although standard methods of marine microalgal dry weight determination are available, these methods did not provide comprehensive details, and the parameters vary among themselves. Without a standard method, a comparison of results among previous studies can be misleading and unreliable. Therefore, the current study aimed to investigate and determine the ideal setting of several parameters in the marine microalgal dry weight determination for laboratory-scale culture. The present findings could assist in developing a standardized protocol to ensure a high quality of biomass for microalgal studies.

**Abstract:**

Microalgal biomass is one of the crucial criteria in microalgal studies. Many reported methods, even the well-established protocol on microalgal dry weight (DW) determination, vary greatly, and reliable comparative assessment amongst published results could be problematic. This study aimed to determine the best condition of critical parameters in marine microalgal DW determination for laboratory-scale culture using four different marine microalgal species. These parameters included the washing process, grades of glass microfiber filter (GMF), GMF pretreatment conditions, washing agent (ammonium formate) concentrations, culture: washing agent ratios (v:v) and washing cycles. GMF grade GF/A with precombustion at 450 °C provided the most satisfactory DW and the highest ash-free dry weight (AFDW)/DW ratio. Furthermore, 0.05 M ammonium formate with 1:2 culture: washing agent ratio and a minimum of two washing cycles appeared to be the best settings of microalgal DW determination. The present treatment increased the AFDW/DW ratio of the four respective microalgae by a minimum of 19%. The findings of this study could serve as a pivotal reference in developing a standardized protocol of marine microalgal DW determination to obtain veracious and reliable marine microalgal DW.

## 1. Introduction

In recent years, microalgae have received considerable attention due to their versatile properties as fertilizer, animal feed, an alternative source of biodiesel feedstock, and human food [1,2,3,4,5]. Microalgal biomass is inarguably one of the main criteria in studying microalgae. However, marine microalgae require additional cleaning procedures in order to obtain precise dry weight (DW). This is because the salt absorbed on the microalgal cell surface or present in the intercellular water could lead to overestimation of the DW [6,7]. Moreover, incomplete removal of salt could intervene in the subsequent downstream processes such as nutritional properties [8,9]. Zhu and Lee [6] studied four different washing agents on marine microalgae and recommended either ammonium formate or ammonium bicarbonate to obtain accurate DW values. Two different approaches frequently utilized for microalgal biomass studies are DW [10,11] and ash-free dry weight (AFDW) [12]. Although AFDW is typically considered as the most accurate predictor for biomass determination [13], DW is often preferred because the process of obtaining AFDW is a time-consuming, expensive, and tedious procedure [14]. In addition, DW serves as an important parameter in estimating biomass concentration, productivity and percentages of cell component [6]. Therefore, an accurate and reliable measurement of DW is essential in documenting the quality of biomass. 

For the DW determination of marine microalgae, the culture suspension is commonly filtered, washed, and dried to a constant weight. Nevertheless, there were large variations in the grades of glass microfiber filter (GMF), GMF pretreatment conditions, washing agent (ammonium formate) concentrations, culture: washing agent ratios (v:v) and washing cycles employed by previous studies (Table 1) in determining the DW of microalgae. Several well-established protocols for determining microalgal biomass were available, but comprehensive details of the marine microalgal DW determination method are lacking, and the parameters of these methods vary among themselves. For example, the protocol of Algal Biomass Organization calls for filtering a specific volume of culture through a pre-weighed Whatman GF/F GMF, washing with ammonium formate (20 g/L for seawater samples), and drying at 105 °C overnight (http://www.algaebiomass.org/wp-content/uploads/2010/06/ABO-Tech-Standards-MDL-V-3.0-FINAL-11-29-10.pdf (accessed on 1 June 2021)). On the other hand, the Application Centre for Renewable Resources method specifies pre-drying the filters at 70 °C for at least 1 day, filtering the 50 mL sample, washing with 50 mL demineralized water, and drying at 105 °C for at least 1 day (https://ixion.bcu.ac.uk/enalgae/assets/pdf/A3/SOP_Dry_weight_biomass_Acrres.pdf%3Bjsessionid=380B92AA5F01C2C0F3E825BED687A744 (accessed on 1 June 2021)). Furthermore, there is no AOAC standard method available for marine microalgal DW determination method. Since there is no standard method for determining the DW of marine microalgal biomass, a comparison of results among previous studies can be misleading and unreliable. In addition, utilization of inappropriate GMF grades and incomplete washing processes could lead to substantial errors in obtaining DW of marine microalgae. Therefore, the present study was conducted to investigate and determine the best setting of several parameters in the determination of marine microalgal biomass DW for laboratory-scale culture using ammonium formate as a washing agent. 

## 2. Materials and Methods

### 2.1. Reagent, Microalgae, and Culture Medium

Ammonium formate (analytical grade from Nacalai Tesque, Japan) was utilized to prepare the washing solution. *Chaetoceros calcitrans* (UPMC-A0010), *Chlorella* sp. (UPMC-A0075), and *Isochyrsis galbana* (UPMC-A0083) were isolated and identified previously [22]. *Nannochloropsis* sp. (UPMC-A0089) was a collection culture from the International Institute of Aquaculture and Aquatic Sciences (I-AQUAS). These microalgae were cultured in sterilized seawater (adjusted to approximately 30 ppt) enriched with Conway medium [(1) trace metal solution (TMS): 21 g/L ZnCl_2_, 20 g/L CoCl_2_.6H_2_O, 9 g/L (NH_4_)_6_Mo_7_O_24_.4H_2_O, 20 g/L CuSO_4_.5H_2_O; (2) vitamin solution: 0.1 g/L vitamin B12, 0.1 g/L vitamin B1, 0.002 g/L vitamin H; (3) Nutrient solution: 1.3 g/L FeCl_3_.6H_2_O, 0.36 g/L MnCl_2_.4H_2_O, 33.6 g/L H_3_BO_3_, 45.0 g/L Na_2_-EDTA, 20 g/L NaH_2_PO_4_.2H_2_O, 100 g/L NaNO_3_, 60 g/L Na_2_SiO_3_.9H_2_O] and maintained in the Aquatic Animal Health and Therapeutics Laboratory, Institute of Bioscience, Universiti Putra Malaysia (UPM). These cultures were incubated at 25 °C under continuous shaking at 150 rpm and illuminated with fluorescent light of 100 µmol photons m^−2^ s^−1^ following 12:12 light/dark cycle.

### 2.2. Total Dry Weight, Ash-Free Dry Weight Determination, and Assessment of Dry Weight

Prior to the study, a total of 10 mL double distilled water was filtered through the GMFs (Whatman, 47 mm) for pretreatment purposes. These filters were combusted under 450 °C for 2 h, cooled in a vacuum desiccator, and weighed. After that, a total of 5 mL microalgal culture was filtered through these GMFs. Next, 5 mL 0.5 M ammonium formate (washing agent) was applied after the culture suspension was filtered through the precombusted filters. For each rinse, the source of vacuum was stopped to ensure the culture/solution covered the filter, and then a suction was employed promptly to remove the culture/solution. The filters were dried at 100 °C for 4 h, cooled in a vacuum desiccator, and then weighed. After that, these oven-dried filters were ashed in a furnace at 540 °C for 4 h, cooled in a vacuum desiccator, and weighed to obtain the AFDW. At least 1 × 10^6^ cells/mL of each microalga were utilized in this study. The assessment of DW was performed through a conventional approach based on 7 parameters: washing process (with and without), GMF (Whatman grade GF/A, GF/C, and GF/F, 47 mm), pretreatment of GMF on different temperatures (dried at 90 °C for 8 h, dried at 100 °C for 8 h, combust at 450 °C for 8 h), pretreatment of GMF at different exposure periods (450 °C for 2 h, 8 h, 16 h, 24 h), culture: washing agent ratios (1:0.5, 1:1, 1:2, 1:4, 1:8 and 1:10) (v:v) and concentrations of washing agent (0.05 M, 0.1 M, 0.5 M, 1 M, 2 M), washing cycles (one, two and three). Only one parameter was changed during each experiment, while the other parameters remained the same. All assays were performed in triplicates.

### 2.3. Statistical Analyses

The collected data were first analyzed using the test of normality. A total of 4 marine microalgal species were utilized to validate the effectiveness of the present treatments on marine microalgal biomass determination. A *t*-test was performed to evaluate the effect of the washing process (ammonium formate) on marine microalgal biomass. One-way analysis of variance (ANOVA) and Tukey’s range post hoc test was conducted to assess the current treatments in determining each marine microalgal biomass separately. The data were analyzed using Minitab 17.1 (Minitab Inc., State College, PA, USA). In all cases, the differences were accepted as significant when *p* < 0.05. 

## 3. Results

### 3.1. Washing Process Yielded Lower Microalgal Dry Weight and Higher AFDW/DW Ratio

**Figure 1:**
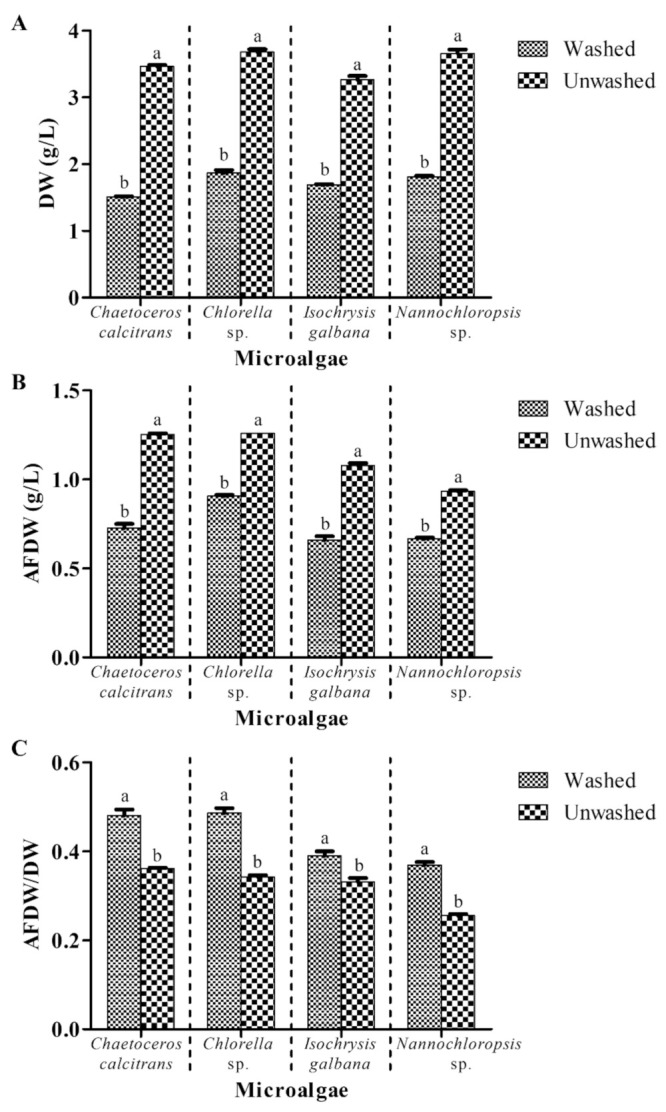
Biomass of four different marine microalgae subjected to ammonium formate (washed) and without ammonium formate (unwashed) treatments. (**A**) dry weight (**B**) ash-free dry weight (**C**) ash-free dry weight/dry weight ratio. The other parameters were unchanged (GF/C; pretreatment of GMF at 450 °C for 8 h; 0.5 M ammonium formate; 1:1 culture: washing agent ratio; one washing cycle). Different letters for the respective microalgae indicate a statistically significant difference (*p* < 0.05) in terms of biomass among the washed and unwashed microalgae. DW, dry weight; AFDW, ash-free dry weight. Data are expressed as mean ± standard error (n = 3).

The initial experiment examined the effect of washing and without the washing process on the microalgal biomass, while the other parameters were unchanged (GF/C; pretreatment of GMF at 450 °C for 8 h; 0.5 M ammonium formate; 1:1 culture: washing agent ratio; one washing cycle). The DW of the four washed microalgae ranged from 1.51 g/L–1.87 g/L, while the DW of the four unwashed microalgae fluctuated from 3.27 g/L–3.68 g/L (Figure 1A). The DW of the four unwashed microalgae was about two times higher (significant at *p* < 0.05) than the corresponding microalgal species that were washed with ammonium formate. 

For the AFDW, a range of 0.66 g/L–0.91 g/L was observed in the four washed microalgae (Figure 1B). Four unwashed microalgae showed an array of 0.93 g/L–1.26 g/L AFDW, which were at least 1.3 times heavier (significant at *p* < 0.05) than the corresponding microalgal species washed by ammonium formate.

*Chaetoceros calcitrans*, *Chlorella* sp., *I. galbana,* and *Nannochloropsis* sp. showed 0.48 ± 0.01, 0.49 ± 0.01, 0.39 ± 0.01, and 0.37 ± 0.01 AFDW/DW ratios, respectively after the washing process (Figure 1C). The washed microalgae demonstrated higher AFDW/DW ratios, which were at least 1.2 times higher (significant at *p* < 0.05) as compared to the corresponding unwashed microalgal species.

### 3.2. Glass Microfiber Filter Grade GF/A Is Recommended for Microalgal Biomass Dry Weight Determination

**Figure 2:**
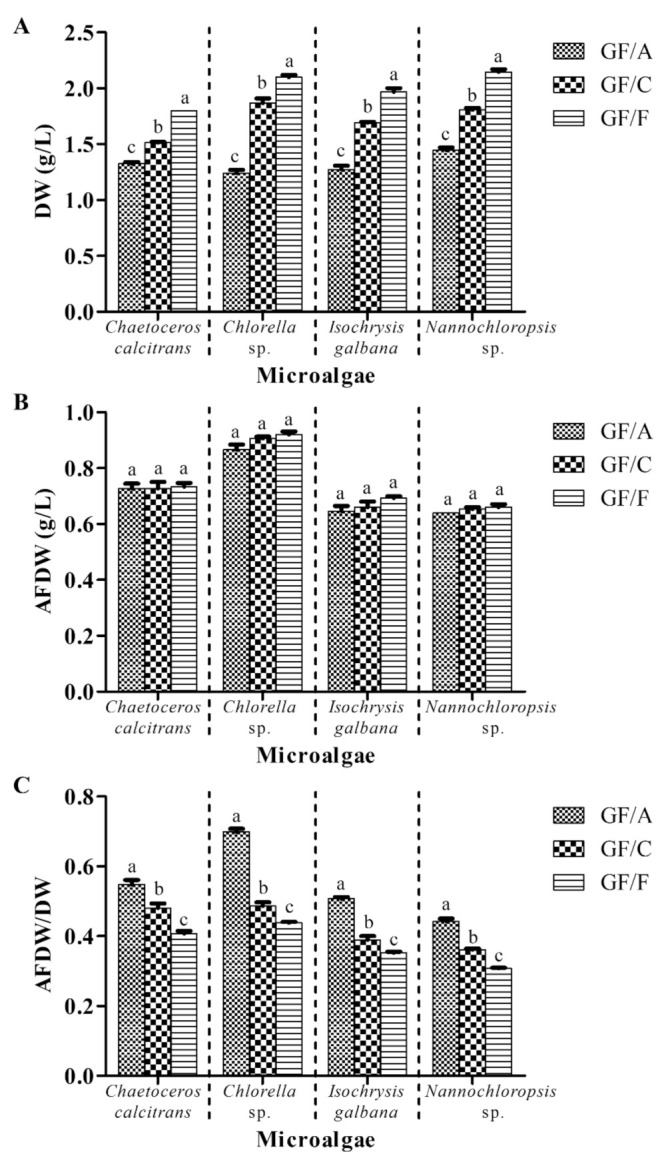
Biomass of four different marine microalgae subjected to different grades of glass microfiber filter. (**A**) dry weight (**B**) ash-free dry weight (**C**) ash-free dry weight/dry weight ratio. The other parameters remained the same (with washing process; pretreatment of GMF at 450 °C for 8 h; 0.5 M ammonium formate; 1:1 culture: washing agent ratio; one washing cycle). Different letters for the respective microalgae indicate statistically significant differences (*p* < 0.05) in terms of biomass among different grades of glass microfiber filter. DW, dry weight; AFDW, ash-free dry weight. Data are expressed as mean ± standard error (n = 3).

This experiment investigated the effect of different grades of GMF on the microalgal biomass, whereas the other parameters remained the same (with washing process; pretreatment of GMF at 450 °C for 8 h; 0.5 M ammonium formate; 1:1 culture: washing agent ratio; one washing cycle). The highest DW of the four marine microalgae was obtained using GMF grade GF/F, followed by GF/C and GF/A (Figure 2A). *Chaetoceros calcitrans*, *Chlorella* sp., *I. galbana* and *Nannochloropsis* sp. demonstrated 1.33 ± 0.01 g/L, 1.24 ± 0.02 g/L, 1.27 ± 0.02 g/L, and 1.45 ± 0.02 g/L, respectively, using GMF grade GF/A. These obtained DW values were significantly lower (*p* < 0.05) than GF/C and GF/F. However, there were no significant differences (*p* > 0.05) in AFDW values amongst all microalgae species using GMF grade GF/A, GF/C, and GF/F (Figure 2B). For AFDW/DW ratio, the highest value was obtained using GMF grade GF/A, followed by GF/C and GF/F for the four microalgae species (Figure 2C). The GMF grade GF/A showed at least 14% higher (significant at *p* < 0.05) AFDW/DW ratios for all microalgae compared to GF/C and GF/F. 

### 3.3. Combustion at 450 °C for Two Hours Is Suggested for the Pretreatment Purpose

#### 3.3.1. Pretreatment of Glass Microfiber Filter at Different Temperatures

**Figure 3:**
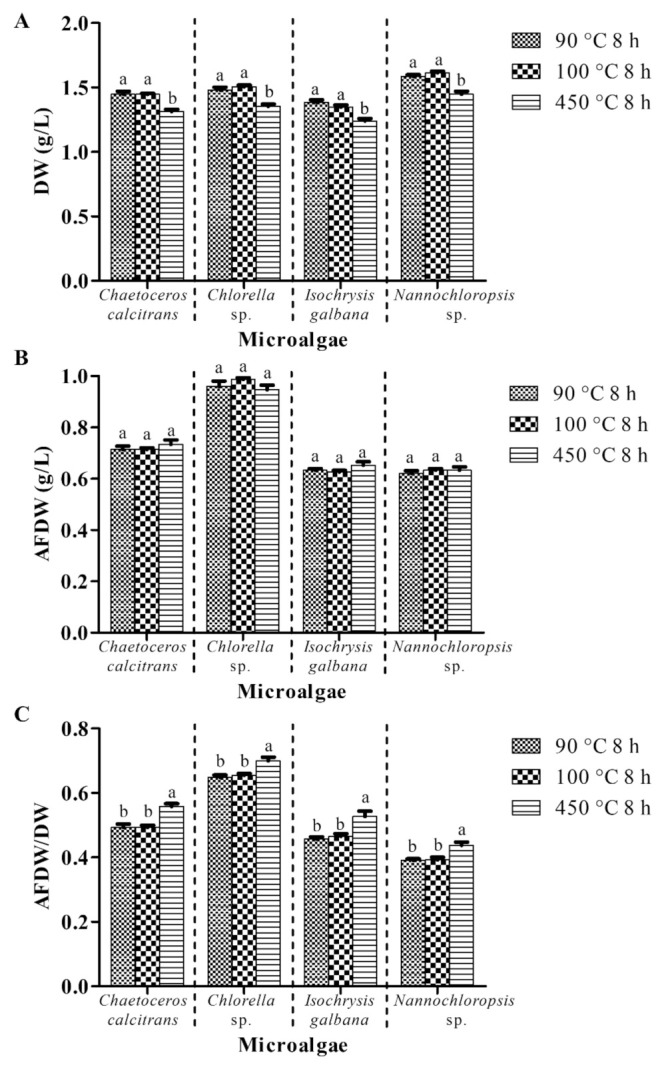
Biomass of four different marine microalgae subjected to the glass microfiber filter that precombusted at different temperatures. (**A**) dry weight (**B**) ash-free dry weight (**C**) ash-free dry weight/dry weight. The other parameters remained constant (with washing process; GF/A; pretreatment of GMF for 8 h; 0.5 M ammonium formate; 1:1 culture: washing agent ratio; one washing cycle). Different letters for the respective microalgae indicate a statistically significant difference (*p* < 0.05) in terms of biomass among the glass microfiber filter that precombusted at different temperatures. DW, dry weight; AFDW, ash-free dry weight. Data are expressed as mean ± standard error of triplicates (n = 3).

This experiment scrutinized the effect of the pretreatment of GMF at different temperatures on the microalgal biomass, whilst the other parameters remained constant (with washing process; GF/A; pretreatment of GMF for 8 h; 0.5 M ammonium formate; 1:1 culture: washing agent ratio; one washing cycle). A range of 1.35 g/L–1.61 g/L DW values for the four microalgae was obtained using the precombusted GMF at 90 °C and 100 °C for 8 h (Figure 3A). Pretreatment of GMF at 450 °C for 8 h yielded around 1.24 g/L–1.45 g/L DW of the four microalgae, which were significantly lower (*p* < 0.05) than those at 90 °C and 100 °C for the respective microalgae. 

All the pretreatments provided an average of 0.72 ± 0.01 g/L, 0.96 ± 0.01 g/L, 0.64 g/L, and 0.63 ± 0.01 g/L AFDW for *C*. *calcitrans*, *Chlorella* sp., *I. galbana,* and *Nannochloropsis* sp., respectively (Figure 3B). The AFDW values of the corresponding microalgal species from pretreatment of GMF at 90 °C, 100 °C, and 450 °C had no significant differences (*p* > 0.05) among different treatments for each species. 

The highest AFDW/DW values of the four microalgae were acquired utilizing the precombusted GMF at 450 °C (Figure 3C). Precombustion at 450 °C yielded about 13%, 7%, 14%, and 12% higher (significant at *p* < 0.05) AFDW/DW ratios for *C*. *calcitrans*, *Chlorella* sp., *I. galbana,* and *Nannochloropsis* sp., respectively compared to preheating at 90 °C and 100 °C. 

#### 3.3.2. Pretreatment of Glass Microfiber Filter at 450 °C for Different Exposure Periods

**Figure 4:**
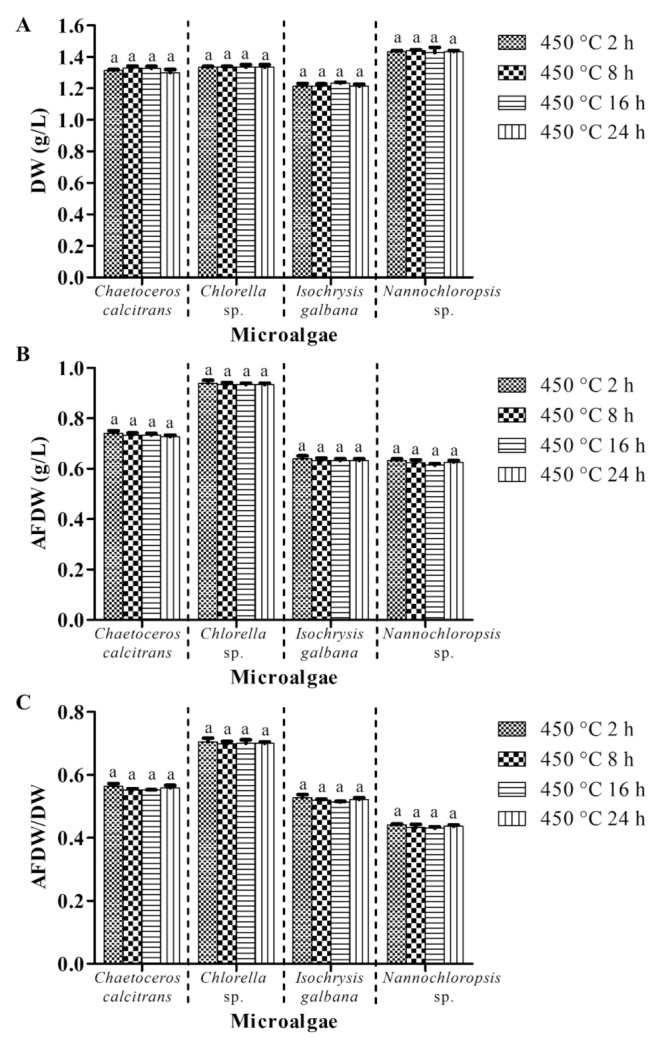
Biomass of four different marine microalgae subjected to glass microfiber filter that precombusted at different exposure periods. (**A**) dry weight (**B**) ash-free dry weight (**C**) ash-free dry weight/dry weight. The other parameters were unchanged (with washing process; GF/A; pretreatment of GMF at 450 °C; 0.5 M ammonium formate; 1:1 culture: washing agent ratio; one washing cycle). Different letters for the respective microalgae indicate statistically significant differences (*p* < 0.05) in terms of biomass among the glass microfiber filters that precombusted at different exposure periods. DW, dry weight; AFDW, ash-free dry weight. Data are expressed as mean ± standard error (n = 3).

This experiment examined the effect of the pretreatment of GMF at 450 °C for different exposure periods on the microalgal biomass, while the other parameters were unchanged (with washing process; GF/A; pretreatment of GMF at 450 °C; 0.5 M ammonium formate; 1:1 culture: washing agent ratio; one washing cycle). Dry weight values of the four microalgae filtered using GMFs that were precombusted at 450 °C for 2 h, 8h 16 h, and 24 h were not significantly different (*p* > 0.05, Figure 4A). Comparable AFDW values with no significant differences (*p* > 0.05) were also observed for all microalgae species (Figure 4B). Similarly, there were no significant differences (*p* > 0.05) in AFDW/DW ratios for all microalgae species using the precombustion at 450 °C at 2 h, 8 h, 16 h, and 24 h (Figure 4C).

### 3.4. A Low Concentration Ammonium Formate (0.05 M) Is Sufficient to Remove Salts from Marine Microalgae

**Figure 5:**
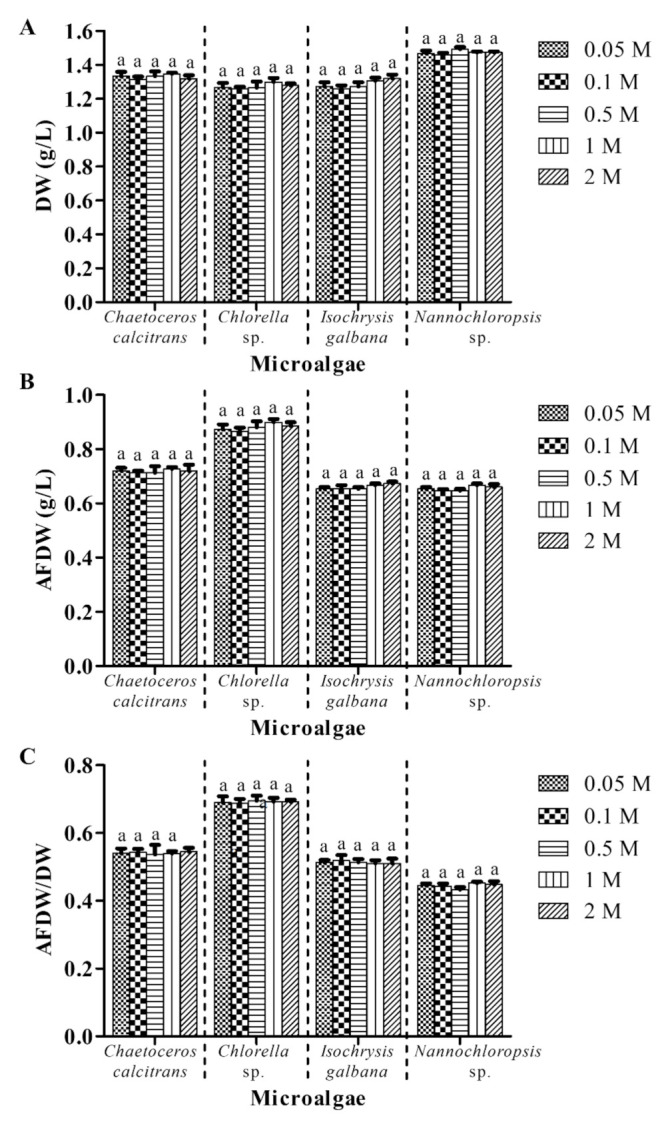
Biomass of four different marine microalgae subjected to different concentrations of ammonium formate. (**A**) dry weight (**B**) ash-free dry weight (**C**) ash-free dry weight/dry weight. The other parameters were the same (with washing process; GF/A; pretreatment of GMF at 450 °C for 2 h; 1:1 culture: washing agent ratio; one washing cycle). Different letters for the respective microalgae indicate statistically significant differences (*p* < 0.05) in terms of biomass among different concentrations of ammonium formate. DW, dry weight; AFDW, ash-free dry weight. Data are expressed as mean ± standard error (n = 3).

This experiment investigated the effect of different concentrations of ammonium formate on the microalgal biomass, whereas the other parameters were the same (with washing process; GF/A; pretreatment of GMF at 450 °C for 2 h; 1:1 culture: washing agent ratio; one washing cycle). Utilization of different concentrations of ammonium formate (0.05 M–2 M) resulted in mean values of 1.33 ± 0.01 g/L, 1.27 ± 0.01 g/L, 1.28 ± 0.01 g/L and 1.47 ± 0.01 g/L DW for *C*. *calcitrans*, *Chlorella* sp., *I. galbana,* and *Nannochloropsis* sp., respectively (Figure 5A). No significant differences (*p* > 0.05) were observed in the DW for all microalgae species. Variations in AFDW values among different ammonium formate concentrations also were not significantly different (*p* > 0.05) in all microalgae species (Figure 5B). In fact, different ammonium formate concentrations demonstrated no significant differences (*p* > 0.05) in AFDW/DW ratios for all four microalgae species (Figure 5C).

### 3.5. Utilization of 1:2 Culture: Washing Agent Ratio (v:v) Is Adequate to Provide Accurate Dry Weight for Marine Microalgae

**Figure 6:**
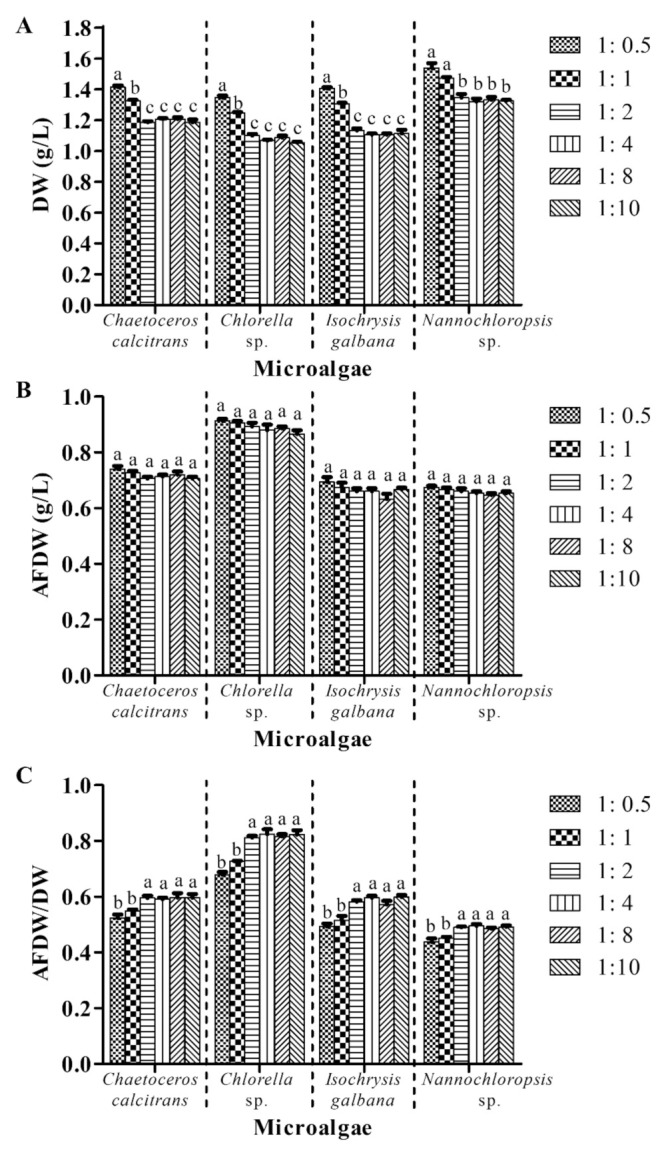
Biomass of four different marine microalgae subjected to different culture: washing agent ratios (v:v). (**A**) dry weight (**B**) ash-free dry weight (**C**) ash-free dry weight/dry weight. The other parameters were unchanged (with washing process; GF/A; pretreatment of GMF at 450 °C for 2 h; 0.05 M ammonium formate; one washing cycle). Different letters for the respective microalgae indicate statistically significant differences (*p* < 0.05) in terms of biomass among different culture: washing agent ratios (v:v). DW, dry weight; AFDW, ash-free dry weight. Data are expressed as mean ± standard error (n = 3).

This experiment scrutinized the effect of different culture: washing agent ratios (v:v) on the microalgal biomass, while the other parameters were unchanged (with washing process; GF/A; pretreatment of GMF at 450 °C for 2 h; 0.05 M ammonium formate; one washing cycle). Utilization of 1:2, 1:4, 1:8 and 1:10 culture: washing agent ratios resulted in significantly lower (*p* < 0.05) DW for the four respective microalgae compared to 1:0.5 and 1:1 culture: washing agent ratios (Figure 6A). No significant differences (*p* > 0.05) of microalgae DW values were found using 1:2, 1:4, 1:8, and 1:10 culture: washing agent ratios. In addition, no significant differences (*p* > 0.05) were detected for AFDW values of the four respective microalgae using different culture: washing agent ratios (Figure 6B). 

Use of 1:0.5 and 1:1 culture: washing agent ratios on the four microalgae demonstrated a range of 0.44–0.73 AFDW/DW ratios, whereas 1:2, 1:4, 1:8, and 1:10 culture: washing agent ratios displayed higher AFDW/DW ratios of 0.49–0.82 (Figure 6C). The AFDW/DW ratios of all microalgae were significantly higher (*p* < 0.05) when using 1:2, 1:4, 1:8, and 1:10 culture: washing agent ratios compared to 1:0.5 and 1:1 culture: washing agent ratios. However, no significant differences (*p* > 0.05) of AFDW/DW ratios were detected among treatments with 1:2, 1:4, 1:8, and 1:10 culture: washing agent ratios.

### 3.6. Two Cycles of Washing Process Are Proposed for Microalgal Biomass Dry Weight Determination

**Figure 7:**
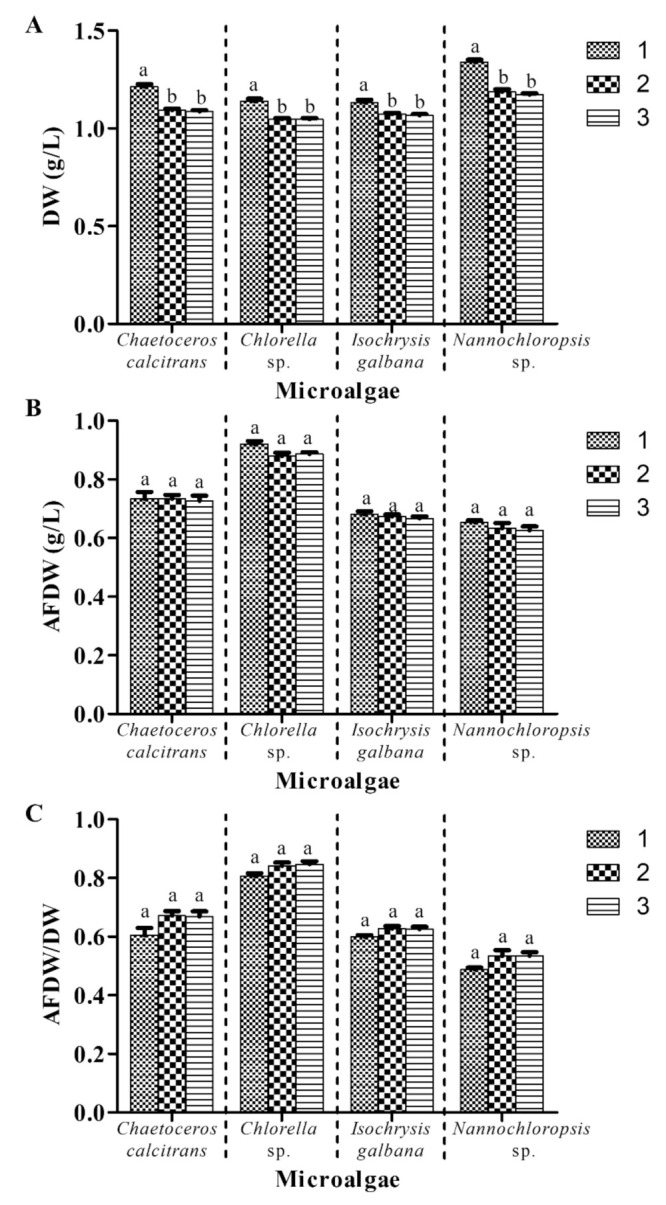
Biomass of four different marine microalgae subjected to a different number of washing cycles. (**A**) dry weight (**B**) ash-free dry weight (**C**) ash-free dry weight/dry weight. The other parameters remained constant (with washing process; GF/A; pretreatment of GMF at 450 °C for 2 h; 0.05 M ammonium formate; 1:2 culture: washing agent ratio). Different letters for the respective microalgae indicate statistically significant differences (*p* < 0.05) in terms of dry weight among different washing cycles. DW, dry weight; AFDW, ash-free dry weight. Data are expressed as mean ± standard error (n = 3).

This experiment examined the effect of the different number of washing cycles on the microalgal biomass, whilst the other parameters remained constant (with washing process; GF/A; pretreatment of GMF at 450 °C for 2 h; 0.05 M ammonium formate; 1:2 culture: washing agent ratio). One washing cycle yielded mean values ranging from 1.13 g/L to 1.34 g/L DW for the four microalgae species (Figure 7A). On the other hand, two and three washing cycles resulted in significantly lower mean values ranging from 1.05 g/L to 1.18 ± 0.01 g/L compared to those subjected to one cycle. There were no significant differences (*p* > 0.05) in the microalgal DW using two and three washing cycles. 

For AFDW, *C. calcitrans*, *Chlorella* sp., *I. galbana,* and *Nannochloropsis* sp. produced mean values of 0.73 ± 0.00 g/L, 0.90 ± 0.01 g/L, 0.67 ± 0.00 g/L and 0.64 ± 0.01 g/L, respectively, using different washing cycles (Figure 7B). However, no significant differences (*p* > 0.05) in AFDW values were observed for each of the microalgae species. Similarly, no significant differences (*p* > 0.05) of AFDW/DW ratios were observed for the four microalgae using different washing cycles (Figure 7C). 

### 3.7. Drying Period of Microalgal Dry Weight

**Figure 8:**
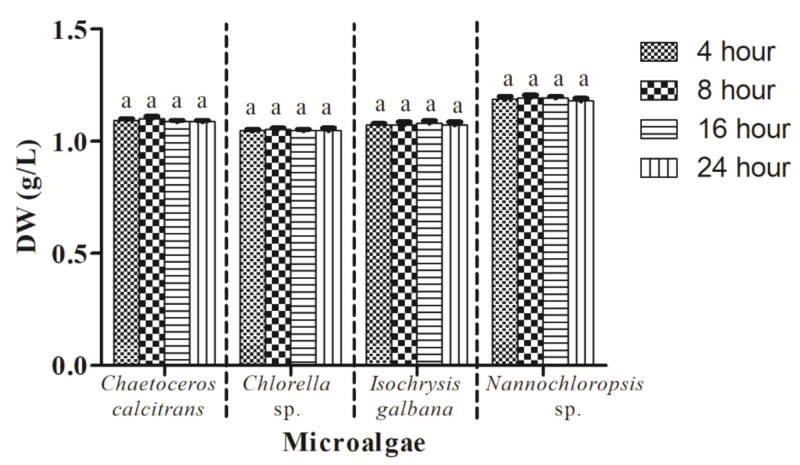
Dry weight of four different marine microalgae subjected to different drying periods at 100 °C. The other parameters remained constant (with washing process; GF/A; pretreatment of GMF at 450 °C for 2 h; 0.05 M ammonium formate; 1:2 culture: washing agent ratio; two washing cycles). Different letters for the respective microalgae indicate statistically significant differences (*p* < 0.05) in terms of dry weight among different drying periods. DW, dry weight. Data are expressed as mean ± standard error (n = 3).

This experiment tested the effect of the four different drying periods (4 h, 8 h, 16 h, and 24 h) at 100 °C on the current microalgal dry weight. A similar dry weight of each microalgae species was obtained using the four different drying periods (Figure 8). In addition, no significant differences (*p* > 0.05) in DW were observed for each of the microalgae species when utilizing these drying periods.

## 4. Discussion

Biomass determination is indispensable in microalgae studies as it is a crucial parameter in estimating biomass concentration, productivity, and percentages of particular cell components [10,11]. Yet, various settings of several parameters in microalgal DW determination have been utilized in previous studies (Table 1), and accurate comparison is almost impossible. Hence, there is a need to examine these parameters to obtain microalgal DW precisely. Since AFDW is regarded as the most accurate measurement of the biomass of an organism [13], the use of the AFDW/DW ratio could determine the reliability of the microalgal DW. However, the process of obtaining AFDW is time-consuming, expensive, and troublesome, thus DW is frequently favored. 

Four marine microalgae, namely *C. calcitrans*, *Chlorella* sp., *I. galbana,* and *Nannochloropsis* sp. were selected in the present study due to their different sizes [22]. In the current study, washed marine microalgae showed relatively lower biomass values in terms of DW and AFDW and a higher AFDW/DW ratio compared to the unwashed microalgae (Figure 1). Results showed that ammonium formate was able to remove salts from marine microalgae effectively in this study, which was similar to results reported by Zhu and Lee [6]. In addition, different grades of GMF displayed distinct retention efficiencies in microalgae. GMF grade GF/F showed the highest DW for the four microalgae in the present study (Figure 2). The nominal rating of GF/A is the largest (1.6 µm), followed by GF/C (1.2 µm) and GF/F (0.7 µm) (www.membrane-solutions.com (accessed on 2 April 2021)). Thus, it was expected that the smallest nominal rating of GMF grade GF/F would retain the most microalgae biomass. However, there were no significant differences (*p* > 0.05) of AFDW values observed for different GMF grades (Figure 2B). The possible explanation could be the microalgal cells might have ‘filled up’ and blocked the smallest pore size of the GMF grade GF/F. This condition might interfere with the washing process, thus overestimating the microalgal DW biomass. The similar AFDW values were probably due to the combustion at 540 °C could eliminate most of the moderate volatile compounds in the marine microalgae. In all the four species, microalgae samples filtered with GMF grade GF/A yielded the highest AFDW/DW ratio compared to the other GMF grades (Figure 2C), indicating small differences between AFDW and DW values when using GMF grade GF/A. Taken together, these results suggested GMF grade GF/A is suitable for microalgal DW determination. 

Pretreatment of GMF (i.e., rinsing with double distilled water and combusting at a particular temperature) is vital as foreign particles might present on the surface of the filter. The pretreatment process could remove the soluble impurities and also minimize the clogging problem [23]. GMF is made of borosilicate glass, which can maintain its properties up to 500 °C (www.membrane-solutions.com, accessed on 2 April 2021). Therefore, the precombustion in this study was performed under 500 °C. Precombustion at 90 °C and 100 °C demonstrated significantly higher (*p* < 0.05) DW values than the precombustion at 450 °C (Figure 3A). Nevertheless, AFDW values of the four respective microalgae were almost the same with no significant difference (*p* > 0.05) for all precombustion conditions. This implies that precombustion at 90 °C and 100 °C might be insufficient either to eradicate the impurities or reduce the clogging problem for the pretreatment. For instance, a temperature such as 450 °C has been reported to be adequate to remove carbon and nitrogen contamination from filters [24]. This observation was also supported by the similar AFDW levels for all microalgae using the present precombustion conditions (Figure 3B). The high temperature of the ashing process eliminated the incomplete combustion problem as comparable values of AFDW of the microalgae were obtained. A significantly higher (*p* < 0.05) AFDW/DW ratio was obtained using precombustion at 450 °C compared to 90 °C and 100 °C (Figure 3C). In addition, no significant difference of microalgae DW, AFDW and AFDW/DW using GMF that was precombusted at 450 °C for 2 h, 8 h, 16 h, and 24 h (Figure 4). Considering the economic aspect, the current study recommended precombustion at 450 °C for 2 h as the ideal pretreatment process.

The washing process is a requisite step in marine microalgal biomass determination as salts may present on microalgae surfaces or in water columns. Ammonium formate has been suggested to be one of the washing agents and widely utilized in removing the salts of marine microalgae [6,25,26,27]. The common concentration of ammonium formate that is used to clean the marine microalgae is 0.5 M (Table 1). In the present study, comparable amounts in terms of DW, AFDW, and AFDW/DW of the four respective microalgae were obtained using different concentrations of ammonium formate (Figure 5). These results suggested that the lowest concentration of ammonium formate (0.05 M) is sufficient for microalgae biomass determination. A previous study showed that a low concentration of ammonium formate (0.15 M) was able to counteract the salt suppression issue in Time-of-Flight-Secondary Ion Mass Spectrometry (TOF-SIMS) [28]. In addition, another similar washing agent, ammonium acetate with 0.15 M was reported to be the best washing solution that removed the salt crystals and other interferences entirely from TOF-SIMS spectra and total ion images [29]. However, the mechanism of ammonium formate in removing salts from marine samples remains obscure. To our knowledge, no study has reported the effect of ammonium formate concentration on the washing process of marine microalgae. Based on the economic aspect, utilization of 0.05 M ammonium formate could reduce the cost by approximately 10-fold compared to the commonly used 0.5 M ammonium formate. Therefore, 0.05 M ammonium formate is proposed for microalgae DW determination. 

Different culture: washing agent ratios were utilized in obtaining the microalgal biomass (Table 1). Two conspicuous groups based on biomass (DW and AFDW/DW) were observed (Figure 6). Group 1 (1:0.5 and 1:1 culture: washing agent ratios) provided higher DW values and lower AFDW/DW ratios for all the four microalgae compared to group 2 (1:2, 1:4; 1:6; 1:10 culture: washing agent ratios). However, no significant differences were noticed for microalgal AFDW for the two groups. This could be due to inadequate volume of washing agent (ammonium formate) to remove salts from microalgae biomass and thus, accounted for the higher DW in group 1. Similar to the ammonium formate concentration, no study was reported on the effect of different culture: washing agent ratios on the washing process of marine microalgae. For AFDW/DW ratio, group 2 demonstrated a significantly higher value than group 1. In addition, the AFDW/DW ratio of group 2 showed at least 6% improvement compared to the previous three treatments (GMF grades, pretreatment conditions, and ammonium formate concentrations). Thus, it is suggested that at least 1:2 culture: washing agent ratio is required to obtain an accurate microalgae DW. 

One to three cycles of the washing process were observed in the previous studies for the microalgal biomass determination (Table 1). Although ammonium formate could eliminate salts from microalgae cells, one cycle washing process was insufficient to completely remove all impurities. This study showed that all microalgae DW values using one washing cycle were significantly heavier than those subjected to two and three washing cycles (Figure 7A). The higher DW level might be due to the presence of salt residues in microalgae after washing. This is supported by Guimarães et al. [27] study, in which the first washing step only removed 93.8% Na and 69.4% K concentrations. Comparable AFDW values of the four microalgae obtained using three different washing cycles in the present study further supported the fact that one cycle is not enough to remove salt contents from marine microalgae. This was similar to the previous study, in which more cycles were needed to effectively remove harmful metals from soil using Na_2_EDTA as a washing agent [30]. Two and three washing cycles yielded higher microalgae AFDW/DW ratios compared to one cycle washing. By increasing the washing cycle, this further improved the AFDW/DW ratio by 3.54–12.65% compared to previous treatments. The increased AFDW/DW ratio indicated the success of pretreatments in the present study in reducing differences between DW and AFDW values. Hence, this result promotes the utilization of at least two washing cycles for precise microalgae DW determination. 

Drying of GMF with samples was often performed at a particular temperature level (ranging from 60 °C to 100 °C) until a constant weight was obtained [6,31,32]. In this study, the drying of GMF with samples was performed at 100 °C for 4 h. The drying process is crucial at this point, as an incomplete drying process might lead to the overestimation of DW. Hence, the present study also adopted the drying process at 100 °C for 8, 16, and 24 h. However, no significant differences (*p* > 0.05) of DW values were found using different exposure periods in this study, indicating that 100 °C for 4 h is enough to acquire accurate marine microalgae DW in the present study. 

This suggested protocol has several limitations. First, this protocol examined microalgal culture with 30 ppt salinity. If microalgae culture with higher salinity (>30 ppt) was used, more washing cycles will be recommended to acquire a more accurate DW. Second, this protocol is only suitable for laboratory scale culture. This is because large-scale microalgal cultivation, especially outdoor open systems, culture could be contaminated with foreign particles (i.e., sand, dust, etc.) that could be larger than the pore size of the filter and lead to the error in DW determination. 

## 5. Conclusions

Microalgal biomass is an important indicator of growth, productivity, and expression of particular cell components. This study showed that the washing process, GMF grades, GMF pretreatment conditions, washing agent concentrations, culture: washing agent ratios, and washing cycles are important factors in determining the reliable DW of marine microalgae. The best conditions for marine microalgal biomass DW determination were: GMF grade GF/A with precombustion at 450 °C for 2 h, 0.05 M ammonium formate as a washing agent, 1:2 culture: washing agent ratios (v:v) and a minimum of two washing cycles. GMF grade GF/A was sufficient for the DW analysis compared to the commonly used GMF grade GF/C and GF/F. Besides, the lowest concentration of washing agent (0.05 M) was adequate to clean marine microalgae with 30 ppt salinity. These pretreatments could reduce the analysis cost and provide reliable DW values of marine microalgae. These findings could aid in developing a standardized protocol for the determination of marine microalgal DW. 

## Figures and Tables

**Table 1:** Different conditions of several critical parameters in marine microalgal biomass dry weight determination adopted in previous studies.

Glass Microfiber Filter Grade	Pretreatment (Combustion)	Concentration of Ammonium Formate (M)	Culture: Ammonium Formate Ratio (v:v)	Cycle of Washing Process	Drying Period (hour)	Drying Temperature (°C)	Reference
GF/F	450 °C, 2 h	0.5	1:2	1	-	95	[6]
GF/C	90 °C, 4 h	0.65	1:1	1	4	90	[15]
GF/F	450 °C, 2 h	0.5	1:2	2	24	95	[16]
-	100 °C, 4 h	0.5	1:1	1	4	100	[17]
GF/C	-	0.5	1:1	3	Overnight	80	[18]
GF/C	450 °C, 16 h	0.5	1:0.75	1	16	100	[19]
GF/C	450 °C, 16 h	0.5	-	1	16	100	[20]
GF/A	-	0.05	-	1	48	60	[21]

## Data Availability

Not applicable.
